# Endoscopic ultrasound-guided radiofrequency ablation for solid pseudopapillary neoplasm of the pancreas

**DOI:** 10.1055/a-2127-4890

**Published:** 2023-08-21

**Authors:** Antoine Coupier, Tawfik Khoury, Rodica Gincul, Fabien Fumex, Andrea Lisotti, Sarah Leblanc, Bertrand Napoléon

**Affiliations:** 1 Department of Gastroenterology, Hôpital privé Jean Mermoz, Ramsay Santé, Lyon, France; 2 Galilee Medical Center, Gastroenterology, Nahariya, Israel, Azrieli Faculty of Medicine, Bar-Ilan University, Safed, Israel; 3 Gastroenterology Unit, Hospital of Imola, University of Bologna, Bologna, Italy

A solid pseudopapillary neoplasm (SPN) is considered a low-grade malignant neoplasm, more often composed of both solid and cystic components with pseudopapillary areas but predominantly solid in 15 % of cases 1 . It is estimated to account for 1 % to 3 % of all pancreatic tumors 2 . Immunostaining of SPNs for beta-catenin is specific 3 . The natural history of these lesions is unknown, but the malignant potential is demonstrated especially in large lesions. The gold standard therapy is surgical resection. Nonetheless, an alternative such as endoscopic ultrasound-guided radiofrequency ablation (EUS-RFA), which is less invasive 4 , should be discussed, especially for young patients with small lesions 5 . All cases of SPN seen and treated with EUS-RFA between 2018 and 2020 were reviewed (IRB 00010835).

Herein, we report on three women, ages 26, 27, and 63, who had pancreatic head lesions (19, 11, and 20 mm, respectively). The case of the 63-year-old woman is described ( Fig. 1 ). EUS fine needle biopsy (FNB) diagnosed an SPN ( Fig. 2 a, b ). Video 1 demonstrates the initial appearance of the lesion in B mode and contrast harmonic mode. The procedure was successfully performed (four shots) with no remaining vascularization in contrast harmonic mode after RFA. At the 3-month follow-up, EUS evidenced hyperechoic nonvascularized necrotic tissue ( Fig. 3 ). No remaining lesion was seen on magnetic resonance imaging (MRI), computed tomography (CT), and EUS at 1 and 2 years.

**Fig. 1:**
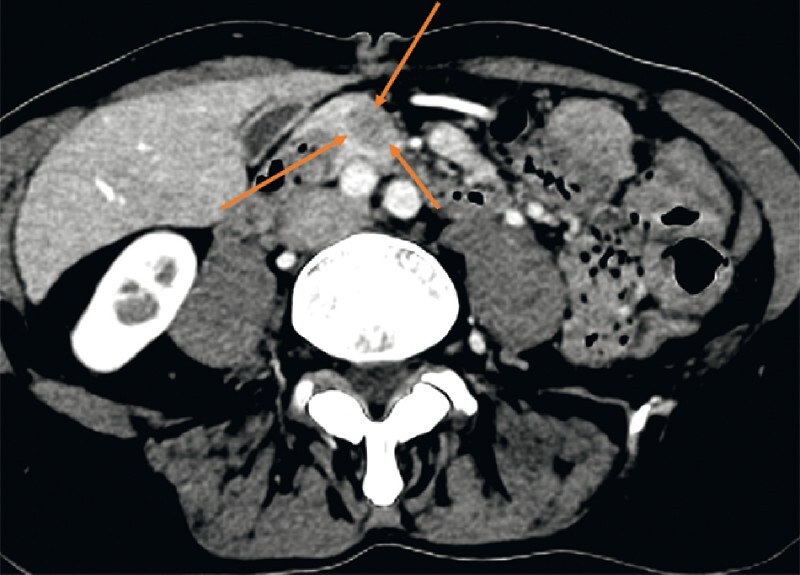
Computed tomography scan showing hypodense pancreatic head lesion (red arrow).

**Fig. 2 a:**
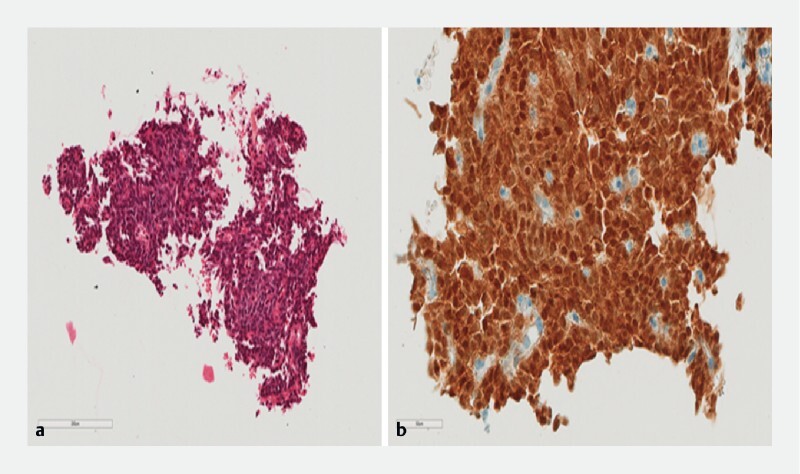
Biopsy showing monomorphic cells on histology. **b** Cells are positively stained for beta-cathenin.

**Video 1** Endoscopic ultrasound-guided radiofrequency ablation for solid pseudopapillary neoplasm of the pancreas.


**Fig. 3:**
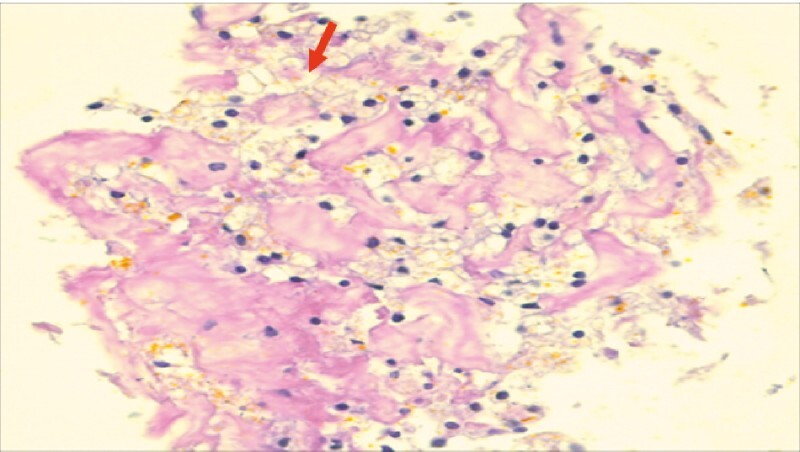
Biopsy showing inflammatory and necrotic cells not stained for beta-cathenin (red arrow).

For the two other cases, one and two RFA sessions were respectively required to completely destroy the lesions. EUS-RFA procedures were uneventful with no post-procedural adverse events. No recurrence was noted at the 24-month follow-up. This treatment option should be considered in patients unfit for pancreatic surgery and could be discussed for small lesions ≤ 2 cm.

Endoscopy_UCTN_Code_TTT_1AS_2AD

## Notes

Endoscopy E-Videos

https://eref.thieme.de/e-videos
*E-Videos* is an open access online section of the journal *Endoscopy* , reporting on interesting cases and new techniques in gastroenterological endoscopy. All papers include a high-quality video and are published with a Creative Commons CC-BY license. Endoscopy E-Videos qualify for HINARI discounts and waivers and eligibility is automatically checked during the submission process. We grant 100% waivers to articles whose corresponding authors are based in Group A countries and 50% waivers to those who are based in Group B countries as classified by Research4Life (see: https://www.research4life.org/access/eligibility/ ). This section has its own submission website at https://mc.manuscriptcentral.com/e-videos
